# Mouse Models of Hepatitis B Virus Infection Comprising Host-Virus Immunologic Interactions

**DOI:** 10.3390/pathogens3020377

**Published:** 2014-04-23

**Authors:** Tadashi Inuzuka, Ken Takahashi, Tsutomu Chiba, Hiroyuki Marusawa

**Affiliations:** Department of Gastroenterology and Hepatology, Graduate School of Medicine, Kyoto University, 54 Shogoin-Kawahara-cho, Sakyo-ku, Kyoto 606-8103, Japan; E-Mails: tinuzuka@kuhp.kyoto-u.ac.jp (T.I.); takaken@kuhp.kyoto-u.ac.jp (K.T.); chiba@kuhp.kyoto-u.ac.jp (T.C.)

**Keywords:** animal model, transgenic mouse, humanized mouse, immune response

## Abstract

Hepatitis B virus (HBV) infection is one of the most prevalent infectious diseases associated with various human liver diseases, including acute, fulminant and chronic hepatitis; liver cirrhosis; and hepatocellular carcinoma. Despite the availability of an HBV vaccine and the development of antiviral therapies, there are still more than 350 million chronically infected people worldwide, approximately 5% of the world population. To understand the virus biology and pathogenesis in HBV-infected patients, several animal models have been developed to mimic hepatic HBV infection and the immune response against HBV, but the narrow host range of HBV infection and lack of a full immune response spectrum in animal models remain significant limitations. Accumulating evidence obtained from studies using a variety of mouse models that recapitulate hepatic HBV infection provides several clues for understanding host-virus immunologic interactions during HBV infection, whereas the determinants of the immune response required for HBV clearance are poorly defined. Therefore, adequate mouse models are urgently needed to elucidate the mechanism of HBV elimination and identify novel targets for antiviral therapies.

## 1. Introduction

Hepatitis B virus (HBV), a member of the Hepadnaviridae family, is an enveloped, circular, single-stranded, and partially double-stranded DNA virus that causes acute and chronic liver disease and hepatocellular carcinoma [1,2]. More than 350 million people worldwide, approximately 5% of the world population, are chronically infected with HBV [3]. Despite our deepening understanding of the pathophysiology of HBV infection, the precise molecular mechanisms of the viral life cycle, persistence of infection, and associated carcinogenesis remain unclear. To understand the pathogenesis caused by HBV infection, adequate animal models that recapitulate HBV-associated liver disease are required. Establishing animal models of HBV infection is difficult as HBV has a narrow host range and exclusively infects humans. Chimpanzees and, to a certain extent, tupaia, the Asian tree shrew, have been used for experimental infection [4,5]. The chimpanzee is the only immunocompetent host fully susceptible to HBV infection, as demonstrated by the induction of acute hepatitis after injection of serum from human HBV carriers [6]. Their large size, associated strong ethical constraints, and the high cost of chimpanzees are increasingly restricting their use for research of human hepatotropic viruses. Although HBV infects tupaias, it causes only mild and transient infection with low viral titers, despite viral DNA replication in the liver, HBsAg secretion into the serum, and the production of antibodies to HBsAg and HBeAg [5]. In addition, tupaias are relatively large animals, difficult to handle in captivity, and not easily available. They are all of outbred origin and their immune systems have not been characterized. Thus, due to the various restrictions for using the currently available models of hepadnavirus infection, and the necessity to work in a well-defined, inbred, and small animal system, most recent developments have focused on mice. Many researchers have attempted to develop mouse models of HBV infection of human livers (Table 1). This review focuses on the history of the currently available mouse models for HBV research to clarify the current status and future directions.

**Table 1:** Comparison of the currently available animal model systems for HBV infection.

Animal model	Advantages	Disadvantages
Human	Natural target of infection	
Chimpanzee	An immunocompetent host fully susceptible to HBV infection, similar to human infection (including cccDNA)	Ethical constraints, large size, high costs, transient infection
Tupaia	Susceptible to HBV infection, similar to human infection (including cccDNA)	Relatively large size, not easily available, outbred animals, transient infection
Mouse	Small, inbred animals, genetically and immunologically well-known	No-infection
Transgenic mouse	convenient, inbred animals, immunological experiment with adoptive transfer	No-infection, immune tolerance
Transfected mouse by hydrodynamic injection	Analysis of mutant strains, immunocompetent	No-infection, transient gene expression,
Transfected mouse by adeno-associated virus	High replication levels, analysis of mutant strains, immunocompetent, relatively long-time gene-expression	No-infection, transient gene expression, possible vector-driven interferences
Human liver-chimeric mouse	Susceptible to HBV infection (including cccDNA), capable to use clinical specimen, assessment of efficacy of anti-HBV agents	high costs, immunocompromised, Transient infection (but relatively long infection)

## 2. Mouse Models

### 2.1. HBV-Transgenic Mouse Models

**Figure 1:**
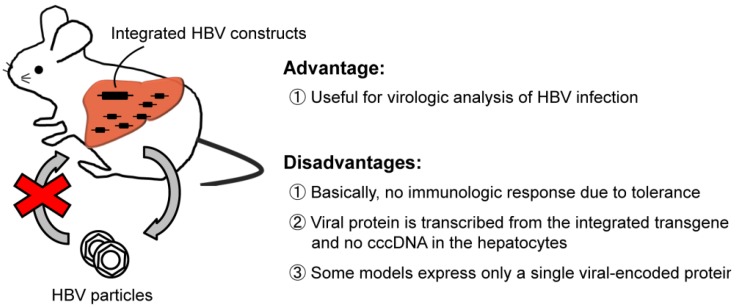
Hepatitis B virus (HBV) transgenic mouse model.

After extensive effort, Chisari *et al.* and other groups developed HBV transgenic mice that express the HBV envelope [7,8,9], core [10,11], precore [12], or X [13,14] gene products. These mice provide the opportunity to analyze heretofore undescribed aspects of HBV virology, such as assembly, transport, secretion, and host immune response to HBV. These models, however, are limited in that they express only a single viral-encoded protein and thus viral replication is not analyzable. To overcome this problem, transgenic mice in which HBV replicates in murine hepatocytes were developed. Araki *et al.* first developed transgenic mice using a construct capable of transcribing all viral genes and observed low levels of HBV replication in the liver as well as the production of HBsAg and HBeAg [15]. Thereafter, transgenic mice with terminally redundant over-length 1.3 HBV-DNA insertion, which produces viral particles at high levels comparable to those of chronic hepatitis patients, were developed [16]. The virions produced in these mice are morphologically indistinguishable from human-derived virions [16] and are infectious when inoculated into chimpanzees [17]. This model, with its advantage of very-high-level HBV replication, provides the opportunity to dissect mechanisms of the viral life cycle and HBV immunobiology, and assess the efficacy of anti-HBV agents (see Figure 1). Although a comprehensive review of the knowledge gained from research using this series of transgenic mice is not possible in this limited review, we present representative studies of these model mice. 

First, studies using HBV transgenic mice have elucidated that HBV is not directly cytopathic for hepatocytes and that both disease pathogenesis and viral clearance are mediated by an antiviral adaptive immune response to HBV [16]. Pathogenic functions of adaptive immunity were demonstrated by the observation that adoptive transfer of HBV-antigen specific cytotoxic T cells (CTLs) to HBV transgenic mice causes acute necroinflammatory liver disease in these mice in which HBV replication itself shows no cytopathic effect [18]. The most important finding in this disease model was that antigen-specific CTLs not only cause hepatocellular injury but also noncytopathically inhibit HBV gene expression and viral replication [18]. Viral clearance is completely blocked by antibodies to interferon (IFN)-γ, and tumor necrosis factor (TNF)-α, indicating that these cytokines are responsible for the noncytopathic antiviral effect of CTLs. The importance of CTLs in the disease pathogenesis and viral clearance was further confirmed by studies of HBV-infected chimpanzees [19]. Antibody-mediated depletion of CTLs delays the onset of viral clearance and liver disease until HBV-specific CTLs become detectable again with the decreasing antibody titer. Together, these findings led to the concept that viral clearance during HBV infection is essentially mediated by noncytolytic mechanisms of CTLs and that liver disease caused by cytolytic mechanisms is an unfavorable side effect of CTL activation. In addition, further studies on HBV transgenic mice revealed that the antigen-nonspecific inflammatory cells exacerbate CTL-induced liver immunopathology and that platelets contribute to both liver disease and viral clearance by facilitating the accumulation of CTLs in the inflamed liver, uncovering the highly complex but coordinated nature of host-viral interaction [20,21,22,23,24,25]. Second, HBV transgenic mice are a powerful tool for evaluating the impact of antiviral cytokines or anti-HBV drugs. Indeed, HBV replication is inhibited by IFN-α, IFN-β, or IFN-γ induced by innate or adaptive immune cells [26,27] and the efficacy of nucleoside analogs, lamivudine [28], adefovir dipivoxil [29] and entecavir [30], has been demonstrated in HBV-transgenic mice. Small interfering RNAs (siRNAs) specifically targeting HBV RNA transcripts suppress HBV replication in the HBV transgenic mice [31,32]. Furthermore, 5’-triphosphorylated HBV-specific siRNAs that are capable of activating the retinoid acid-inducible protein I-dependent pathway more efficiently control HBV by the dual mechanisms of direct suppression of the viral gene expression and induction of an intrahepatic type I IFN response [33].

Although no one doubts that HBV transgenic mouse model has greatly expanded our knowledge of hepatitis B, several limitations of this model must be taken into account. First, HBV particles produced by the transgenic mice do not enter into murine hepatocytes, which lack HBV-specific receptors [34]. Researchers thus cannot study the infection step of the HBV life cycle. Second, covalently closed circular DNA (cccDNA) is not detected in the liver of HBV transgenic mice. This cannot be dismissed, because cccDNA, the template of viral transcription in natural infection, could be an important therapeutic target to achieve complete eradication of HBV [35,36]. Finally, due to the immunotolerant nature of HBV transgenic mice, the mice do not develop hepatitis per se and only downstream events after adoptive transfer of *in vitro*-stimulated HBV-antigen specific CTLs are analyzable, which prevents comprehensive understanding of the immune response to HBV. This limitation, however, was partially overcome by the newly developed HBV transgenic mouse model, where T and B cell adaptive immune system was ablated by crossing HBV transgenic mice with *Rag1-*deficient mice [37,38]. In this model, adoptive transfer of HBV-naïve splenocytes to adult but not young transgenic mice resulted in the spontaneous development of effective immune response to HBV with concomitant liver disease. With the advantage that naïve immune system is primed to viral antigen originating in the liver, this model is suitable for the analysis of immune-priming event and has provided the opportunity to dissect the age-dependent immunological differences in HBV clearance and persistence [37,38].

### 2.2. HBV Transfection by Hydrodynamic Injection

**Figure 2:**
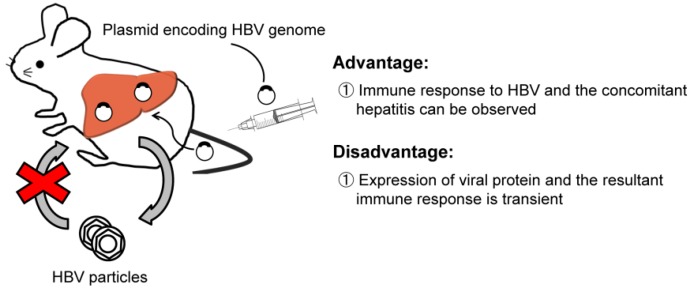
Hydrodynamic injection model.

Because transgenic mice are immunologically tolerant to the virus, it is difficult to study the host immune response and the resultant pathophysiology of HBV infection. To overcome this problem, several researchers applied the transient expression of HBV protein in the liver of adult mice using hydrodynamic injection techniques (see Figure 2) [39,40]. Hydrodynamic injection techniques involve rapid injection of a high volume of fluids containing naked DNA encoding partial or full-length HBV genome sequences into the tail vein of mice. Hydrodynamic injection of a naked plasmid DNA encoding a supergenomic HBV 1.3-length transgene into inbred mice could induce high levels of HBV replication in the liver, producing circulating HBV DNA at levels of 8 × 10^6^ copies/mL blood. HBV replication in the liver, however, is rapidly terminated within 15 days after injection by specific antiviral antibodies and CTLs if the mice are immunocompetent. In contrast, the virus persists over 80 days after hydrodynamic injection in immunodeficient NOD/scid mice lacking functional B-, T-lymphocytes, and natural killer cells [39]. This experimental approach using a panel of immunodeficient mouse strains for the examination of anti-HBV immunologic responses clarified that hepatic clearance of the input HBV templates requires a variety of effectors, including CD4+ and CD8+ T cells, natural killer cells, Fas, IFN-gamma (IFN-γ), IFN-alpha/beta receptor (IFN-α/βR1), and TNF receptor 1 (TNFR1) [41]. On the other hand, B cells and perforin are not essential for clearance of the HBV transcriptional template from the liver in the hydrodynamically-transfected mouse model. Based on those findings, CTLs (CD8+ T cells) are thought to be the key cellular effectors mediating HBV clearance from the liver by a Fas-dependent, but perforin independent, process in which natural killer cells, IFN-γ, TNFR1, and IFN-α/βR play supporting roles, suggesting the existence of redundant pathways that inhibit HBV replication [41]. Thus, at present, in addition to the adaptive immune system like CD4+ and CD8+ T cells, it is thought that the innate immune system also plays an important role in HBV-induced liver inflammation and disease progression [42].

### 2.3. HBV Transfection by Adeno-Associated Virus

Recently, another mouse model of HBV replication was newly developed. Dion *et al.* described a mouse model that allows the HBV persistence based on the liver-targeted transduction of adeno-associated virus serotype 2/8 (AAV2/8), which delivered the HBV genome enabling the study of viral infection for up to one year [43]. In this model, hepatitis B core antigen (HBcAg) expression was detected in approximately 60% of hepatocytes, contrasting with the 5 to 10% of hepatocytes with HBcAg expression achieved by the hydrodynamic injection of conventional plasmids encoding HBV genome. In addition, AAV allows homogeneous transduction of the liver, whereas not all parts of the liver are reached after hydrodynamic injection [39]. This mouse model recapitulates virological and immunological characteristics of chronic HBV infection, and could be useful for the development of new treatment and immune-based therapies or therapeutic vaccines for chronic HBV infections [43,44].

### 2.4. Chimeric Mouse Models of HBV Infection

As described above, HBV transgenic mouse models have had important roles for clarifying the pathophysiology of the host immune response to HBV. Because HBV does not infect the murine hepatocytes, these mice do not recapitulate natural HBV infection. To overcome these problems, researchers have attempted to transplant human hepatocytes into mice. The development of the trimera mouse was one result of such an attempt, in which human hepatocytes were transplanted under the kidney capsule of immunodeficient mice after lethal irradiation [45]. The number of hepatocytes that could survive on the kidney capsule was small, however, and the normal liver architecture was not observed. Although 85% of transplanted mice developed HBV viremia, the titer was less than 10^5^ virus particles or IU/mL and lasted only ~20 days [45].

To establish HBV infection in mice, two human-liver chimeric mouse models were developed. The first was the urokinase-type plasminogen activator (uPA)/scid mouse, which remains the most widely model used for infection studies and preclinical drug evaluation. Transgenic mice in which the urokinase gene expression is driven by the human albumin promoter/enhancer (uPA mice) show accelerated hepatocyte death with consequent chronic hepatocyte growth stimulation [46]. Transplanted rat hepatocytes proliferate and repopulate in the injured livers of immunodeficient uPA mice, which are produced by mating uPA transgenic mice with scid mice (uPA/scid mice) [47]. Human hepatocytes transplanted into uPA/scid mice were demonstrated to successfully proliferate and replace apoptotic murine hepatocytes. [48,49,50]. The disadvantages of uPA/scid mice are infertility and susceptibility to fatal hemorrhaging [46,51]. The second model was a Fah^−/−^Rag2^−/−^Il2rg^−/−^ mouse, deficient in fumarylacetoacetate hydrolase (Fah), recombination activating gene 2 (Rag2), and gamma-chain of the receptor for IL-2 (Il2rg). Fah is the last enzyme in the tyrosine breakdown pathway, and its deficiency leads to liver failure in mice. Treatment with 2-(2-nitro-4-trifluoromethylbenzyol)-cyclohexane-1, 3-dione (NTBC) prevents the accumulation of toxic metabolites and the resultant hepatotoxicity. Induction of liver injury by the withdrawal of NTBC allows for successful transplantation of human hepatocytes with high rates of chimerism [52,53]. Because the mice are immunocompromised following the injection of 1 million human hepatocytes into the mouse spleen, a proportion of the transplanted cells engraft in the liver after migrating via the splenic and portal veins. A few days post-transplantation, small clusters of human hepatocytes begin to proliferate within the mouse liver, forming larger regenerative nodules that eventually merge together and replace the diseased liver parenchyma. The levels of human chimerism can be estimated by measuring the levels of human albumin circulating in mouse serum.

**Figure 3:**
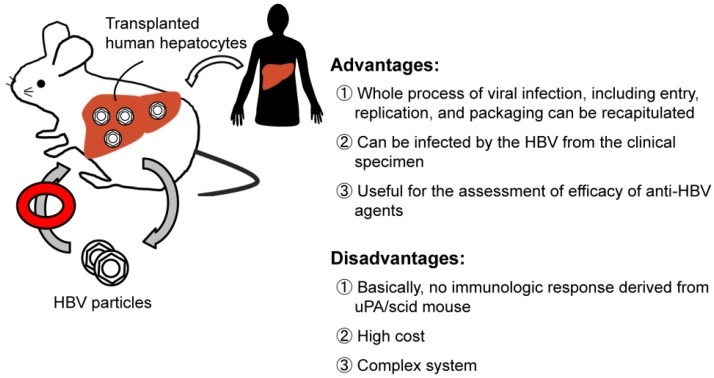
Chimeric mouse model.

These two types of human hepatocyte chimeric mice are susceptible to HBV infections (see Figure 3) [53,54,55]. The establishment of HBV infection is generally first achieved in a small minority of human hepatocytes and several weeks are needed to accomplish viral spreading. After that, nearly all human hepatocytes stain HBcAg-positive and viremia reaches a stable plateau, which directly correlates with the levels of human chimerism [53,54,55,56].

Because human hepatocyte chimeric mice are immunocompromised, they are not suited for vaccine studies or evaluation of immune responses. These mice, however, are a promising tool for evaluation of anti-HBV agents [54,57,58] and of susceptibility of mutant strains to various drugs [59]. More importantly, the largest advantage of the human hepatocyte chimeric mice is that they are the sole model fully recapitulating the genomic maintenance of nuclear HBV cccDNA. Using this model, Belloni *et al* demonstrated that IFN-α suppresses HBV replication through the mechanism of epigenetic control of cccDNA function and transcription [60]. Since cccDNA can be an important therapeutic target to achieve complete eradication of HBV, chimeric mice experiments aiming at elucidating the molecular mechanism whereby cccDNA activity is controlled would help the development of more effective therapeutics.

### 2.5. Genetically Humanized Mice

Murine hepatocytes do not support the entry of HBV and hepatitis C virus (HCV), due to the lack of receptor molecules specific for HBV and HCV. Based on the observation that CD81 and occluding (OCLN) comprise the minimal set of human factors required to render mouse cells permissive to HCV entry [61], Dorner *et al*. showed that either transient or stable expression of these two human genes is sufficient to allow viral uptake and support HCV infection in immunocompetent inbred mice [62,63]. In principle, similar strategy can be applied for the generation of mouse model in which the entire HBV life cycle is recapitulated. However in case of HBV, despite the identification of sodium taurocholate cotransporting polypeptide (NTCP) as a long-sought functional receptor for HBV [34,64,65,66], recent study demonstrated that in mouse hepatocytes NTCP expression allows HBV entry but is not sufficient to support HBV infection, suggesting the existence of murine restriction factors that limit HBV infection [67]. Thus, future studies for the identification of such factors would be required for the development of immunocompetent genetically humanized mice that support HBV infection.

### 2.6. Humanized Mice with Human Immune System and Liver Tissues

Due to the absence of a functional immune system, the above-described uPA/scid and Fah^−/−^Rag2^−/−^Il2rg^−/−^ mouse models support HBV infection but no liver disease is observed [48,53]. To reproduce human immune response to HCV in a small animal model, Washburn *et al.* developed humanized mice reconstituted with human immune system and liver tissues (AFC8-hu HSC/Hep) [68]. They used Balb/C Rag2^−/−^γC-null mice that were genetically engineered to express a fusion protein of FK506 binding protein (FKBP) and caspase 8 with inducible suicidal activity in hepatocytes under the control of albumin promoter (AFC8). Co-transplantation of human CD34+ hematopoietic stem cells and human hepatocyte progenitors into the transgenic mice treated with FKBP dimerizer allowed for the successful engraftment of immune cells and hepatocytes. AFC8-hu HSC/Hep became infected with HCV in the livers, generated a human immune T cell response against the virus, and developed hepatitis and fibrosis [68]. Thus, HBV infection experiments using these mice are expected to uncover heretofore unsuspected virologic and immunologic aspects of HBV infection.

## 3. Conclusions

The history of the fight between HBV and humans began in 1965 when Baruch Blumberg *et al*. discovered the Australia antigen later determined to be HBsAg [69], followed by the discovery of the association between the Australia antigen and specific hepatitis viral infection. After that, many basic and clinical studies have shed light on the virology and pathophysiology of HBV and have attempted to establish mouse models for HBV infection. The mechanisms of a wide range of immune responses against HBV and the resultant clinical phenotypes have not yet been determined. To gain further insight into the host-virus interaction during HBV infection, further progress toward establishing suitable animal models for detailed studies of HBV infection and thus the development of a robust animal model are required.
